# Inner cell mass incarceration in 8-shaped blastocysts does not increase monozygotic twinning in preimplantation genetic diagnosis and screening patients

**DOI:** 10.1371/journal.pone.0190776

**Published:** 2018-01-09

**Authors:** Yi-Fan Gu, Qin-Wei Zhou, Shuo-Ping Zhang, Chang-Fu Lu, Fei Gong, Yue-Qiu Tan, Guang-Xiu Lu, Ge Lin

**Affiliations:** 1 Institute of Reproductive and Stem Cell Engineering, School of Basic Medical Science, Central South University, Changsha, China; 2 Reproductive & Genetic Hospital of CITIC-XIANGYA, Changsha, China; 3 National Engineering and Research Center of Human Stem Cell, Changsha, China; 4 Key Laboratory of Reproductive and Stem Cell Engineering, National Health and Family Planning Commission, Changsha, China; Peking University Third Hospital, CHINA

## Abstract

**Background:**

The use of assisted reproductive technology (ART) has been reported to increase the incidence of monozygotic twinning (MZT) compared with the incidence following natural conception. It has been hypothesized that splitting of the inner cell mass (ICM) through a small zona hole may result in MZT. In this study, using a cohort of patients undergoing preimplantation genetic diagnosis/screening (PGD/PGS), we compared the clinical and neonatal outcomes of human 8-shaped blastocysts hatching with ICM incarceration with partially or fully hatched blastocysts, and attempted to verify whether this phenomenon increases the incidence of MZT pregnancy or negatively impact newborns.

**Methods:**

This retrospective study included 2059 patients undergoing PGD/PGS between March 1, 2013, and December 31, 2015. Clinical and neonatal outcomes were only collected from patients who received a single blastocyst transfer after PGD/PGS (n = 992). A 25- to 30-μm hole was made in the zona of day 3 embryos by laser. The blastocysts were biopsied and vitrified on day 6. The biopsied trophectoderm (TE) cells were analyzed using different genetic methods. One tested blastocyst was thawed and transferred to each patient in the subsequent frozen embryo transfer cycle. All the biopsied blastocysts were divided into three types: 8-shaped with ICM incarceration (type I), partially hatched without ICM incarceration (type II), and fully hatched (type III). ICM/TE grading, clinical and neonatal outcomes were compared between the groups.

**Results:**

The percentage of grade A ICMs in type I blastocysts (22.2%) was comparable to that in type III blastocysts (20.1%) but higher than that in type II blastocysts (4.5%). The percentage of grade A TEs in type I blastocysts (4.2%) was comparable to that in type II (3.6%) but lower than that in type III (13.5%). There were no significant differences in clinical pregnancy, MZT pregnancy, miscarriage, live birth, MZT births, and neonatal outcomes between the groups.

**Conclusions:**

Compared to partially and fully hatched blastocysts, 8-shaped blastocysts with ICM incarceration showed relatively higher ICM and lower TE grades. ICM incarceration in 8-shaped blastocysts does not increase the incidence of MZT and has no negative effects on newborns in PGD/PGS patients.

## Introduction

The global percentage of monozygotic twinning (MZT) following natural in vivo conceptions is approximately 0.4–0.45% [1, 2]. However, its etiology remains to be clarified [3].

The use of assisted reproductive technology (ART) has increased steadily, and in recent years, ART use has been reported to increase the incidence of MZT by 2- to 12-fold compared with the incidence following natural conception [4, 5]. Although this phenomenon has attracted the attention of clinicians due to the association of MZT with a high risk of perinatal mortality and congenital anomalies, very less is known about the reasons for this increased frequency of MZT associated with ART [6]. Current evidence suggests that multiple factors might be responsible, including maternal age, ovarian stimulation, prolonged embryo culture, altered in vitro culture conditions, and zona pellucida manipulation such as intracytoplasmic sperm injection(ICSI) and assisted hatching (AH) [7]. Due to the high incidence of MZT in births that occur after ICSI [8], AH [9], and blastocyst culture [10], it has been hypothesized that splitting of the ICM through a small zona hole may result in MZT [11].

While some studies support this hypothesis, currently available evidence is insufficient. Van Langendonckt et al [12] reported that transferring a human 8-shaped hatching blastocyst with ICM incarceration (ICM trapped in a small zona opening) and another fully expanded blastocyst without hatching resulted in a trichorial triplet pregnancy, but in their study it was unclear which blastocyst the MZT pregnancy originated from. Behr and Miki [13] reported an 8-shaped blastocyst separated into two identical embryos in vitro completely on day 6; however, this is a rare phenomenon that was not reported by other researchers. Recently, Yan et al. [14] reported that extended *in vitro* culture can result in 8-shaped blastocyst hatching at higher frequencies than in vivo fertilization, and this 8-shaped hatching may disturb ICM herniation, leading to increased risk of ICM splitting in mouse blastocysts. Moreover, researchers observed the same phenomenon in human blastocysts [15]. However, researchers have not followed the pregnancy and offspring of these figure 8-shaped blastocysts with ICM splitting; therefore, the relationship between this phenomenon and MZT pregnancies remain to be confirmed.

Thus far, very limited information is available regarding the clinical and neonatal outcomes of human 8-shaped hatching with ICM incarceration, and the relationship between this phenomenon and MZT pregnancy remains unclear. Preimplantation genetic diagnosis/preimplantation genetic screening (PGD/PGS) blastocysts are suitable models to test this hypothesis because it involves ICSI manipulation, AH, and blastocyst culture, all of which are strongly associated with MZT. Furthermore, 8-shaped hatching with ICM incarceration can be easily seen in PGD/PGS blastocysts. In this study, using a cohort of patients undergoing PGD/PGS, we compared the clinical and neonatal outcomes of human 8-shaped blastocysts hatching with ICM incarceration with fully hatched blastocysts, and attempted to verify whether this phenomenon increases the incidence of MZT pregnancy or negatively impact newborns.

## Materials and methods

### Patient selection and study design

**Fig 1:**
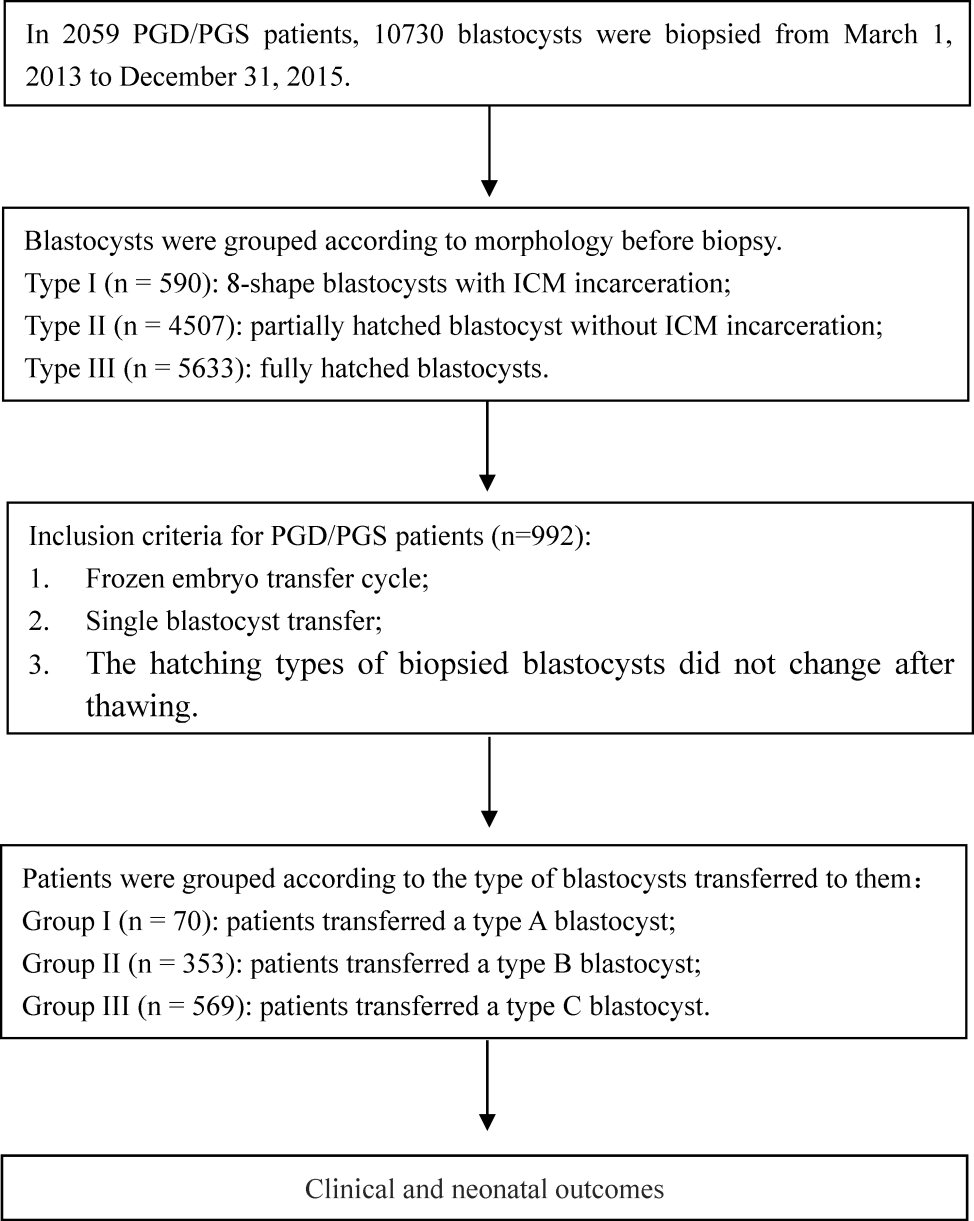
Flowchart of study design.

**Fig 2:**
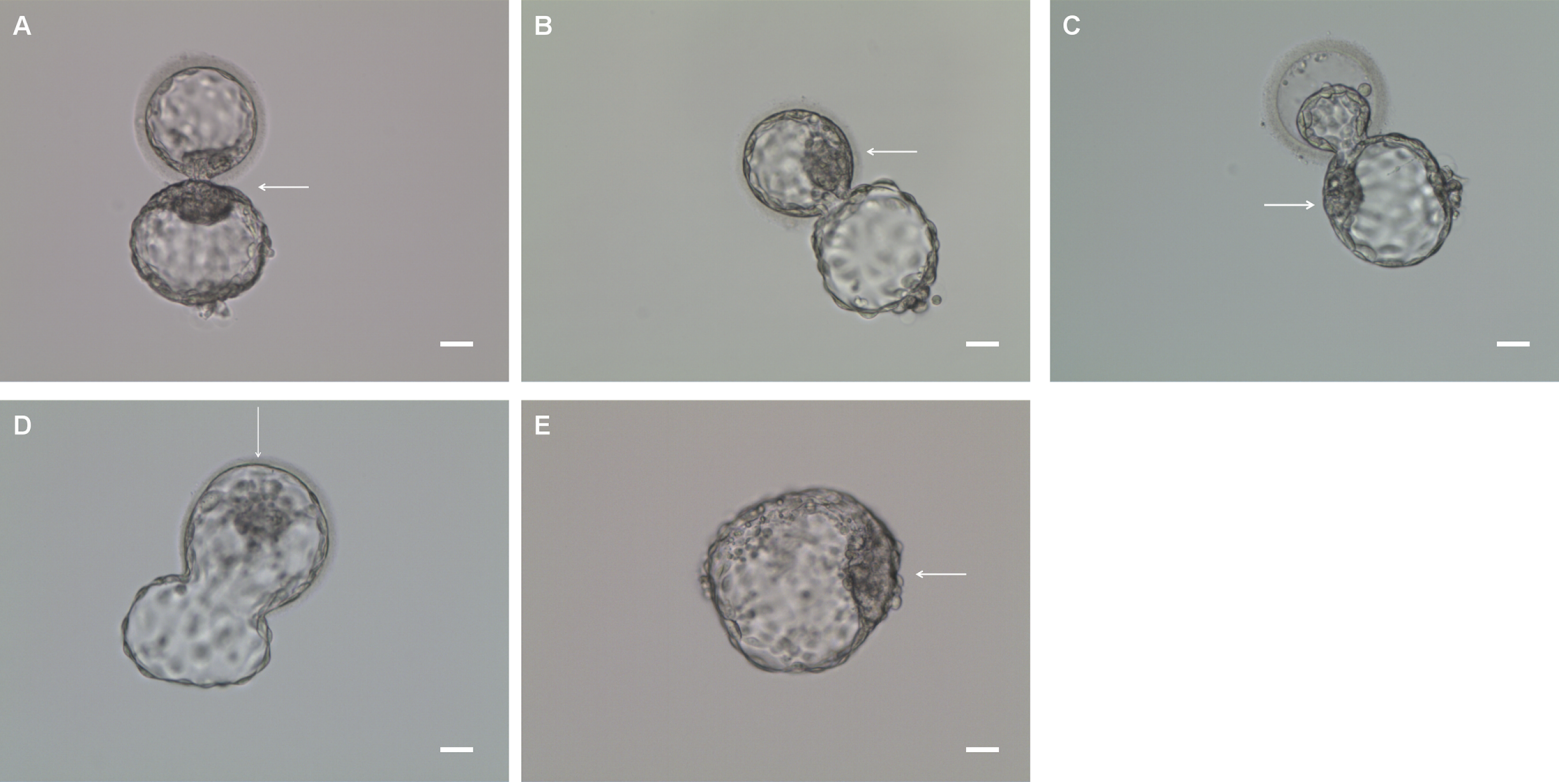
Three hatching types of PGD/PGS blastocysts before biopsy. Type I: Figure 8-shaped blastocysts with ICM incarceration (A) in which a part of TE cells were hatched out, but ICMs were trapped in the ZP opening (zona hole ≤ 30 μm). Typically, ICM cells were split both inside and outside the ZP hole, but remained connected by a narrow bridge. Type II: Partially hatched blastocysts without ICM incarceration in which ICMs were inside (B) or outside(C) of the ZP opening, or the blastocysts hatched with a U-shape(D) (zona hole expanded >30 μm). Type III: Fully hatched blastocysts in which all TE and ICM cells were hatched out of zone(E). Arrow: inner cell mass. Bar = 30 μm.

This retrospective study analyzed the data of 2059 PGD/PGS patients from March 1, 2013, to December 31, 2015, at the Reproductive and Genetic Hospital of CITIC-Xiangya. Fig 1 presents the study design. All biopsied blastocysts were divided into three types: 8-shaped blastocysts with ICM incarceration (type I) (Fig 2A and S1 Fig), partially hatched without ICM incarceration (type II) (Fig 2B, 2C and 2D and S2 Fig), and fully hatched (type III) (Fig 2E and S3 Fig).The clinical and neonatal outcomes were only collected from those patients who received a single blastocyst transfer after PGD/PGS and the hatching types of biopsied blastocysts did not change after thawing. A total of 992 PGD/PGS cycles met our inclusion criteria. The patients were divided into three groups based on blastocyst morphology before embryo transfer: type I blastocysts were transferred to 70 patients (Group I); type II blastocysts, to 353 (Group II); and type III blastocysts, to 569 (Group III). Additionally, we investigated the MZT pregnancy in 15939 artificial insemination patients who underwent natural cycle during the same period as a natural conception control group. The study protocol was approved by the Ethics Committee of CITIC-Xiangya (LL-SC-SG-2014-016).

### In vitro fertilization (IVF) and artificial insemination (AI)

Either a long luteal gonadotropin-releasing hormone (GnRH) agonist protocol or an antagonist protocol was used for ovarian stimulation, as described previously [16]. Briefly, 5,000–10,000 IU of human chorionic gonadotropin (hCG; Pregnyl, Merck) was injected when two-thirds of the follicles reached 18 mm. The oocytes were collected after 34–36 h of hCG administration under transvaginal ultrasound guidance. All oocytes were fertilized by ICSI at 4–6 h after oocyte retrieval, and checked for normal fertilization at 16–18 h after injection. All the embryos were then cultured to blastocyst stage in sequential media (G1 and G2; Vitrolife) in the presence of 6% CO_2_, 5%O_2_, and 89% N_2_ in a mini-incubator (Cook) for further manipulation.

In artificial insemination, all patients underwent natural cycle and did not receive clomiphene citrate or gonadotropins. Intrauterine insemination (IUI) or intracervical insemination (ICI) with husband or donor semen were performed on the day after detection of the luteinizing hormone (LH) surge.

### Embryo biopsy

On day 3, a 25- to 30-μm hole was made in the zona pellucida (ZP) of all the cleaved embryos. On the morning of day 6, the ICM and TE morphology of blastocysts were evaluated according to the criteria described by Gardner and Schoolcraft [17]. Briefly, blastocysts were given a numerical score from 1 to 6 on the basis of their stage of expansion and hatching status, as follows: 1, an early blastocyst with a blastocoel that is less than half of the volume of the embryo; 2, a blastocyst with a blastocoel that is half of or greater than half of the volume of the embryo; 3, a full blastocyst with a blastocoel completely filling the embryo; 4, an expanded blastocyst with a blastocoel volume larger than that of the early embryo, with a thinning zona; 5, a hatching blastocyst with the trophectoderm starting to herniate though the zona; and 6, a hatched blastocyst, in which the blastocyst has completely escaped from the zona. The development of the inner cell mass was assessed as follows: A, tightly packed, many cells; B, loosely grouped, several cells; or C, very few cells. The trophectoderm was assessed as follows: A, many cells forming a cohesive epithelium; B, few cells forming a loose epithelium; or C, very few large cells. Blastocysts in which the ICM grade was grade A or B and had at least a small amount of TE cells (grade A, B, or C) that had herniated out of the ZP opening (blastocyst stage 5 or 6) were chosen for biopsy. Grade C blastocysts or early blastocysts on day 6 were excluded from being biopsied. Blastocysts were positioned using the holding pipette to locate the herniating TE at the 3 o’clock position. Approximately 5–10 TE cells were aspirated with a biopsy pipette (internal diameter, 30 μm) and dissected by laser irradiation (Zilos TK, Hamilton Thorne Biosciences). The TE cells were washed thrice in G-MOPS medium (Vitrolife) and detected using different genetic methods.

All biopsied blastocysts were divided into three types, which was summarized in Table 1.

**Table 1:** Morphological description of three hatching types in PGD/PGS blastocysts.

Hatching type	Morphological description
**I**	Figure 8-shaped blastocysts with ICM incarceration in which a part of TE cells were hatched out, but ICMs were trapped in the ZP opening (zona hole ≤30 μm; Fig 2A and S1 Fig). Typically, ICM cells were split both inside and outside the ZP hole, but remained connected by a narrow bridge.
**II**	Partially hatched blastocysts without ICM incarceration in which ICMs were inside of the ZP opening (Fig 2B and S2A, S2B and S2C Fig)
	Partially hatched blastocysts without ICM incarceration in which ICMs were outside of the ZP opening (Fig 2C and S2D, S2E and S2F Fig)
	U-shaped blastocysts without ICM incarceration (zona hole expanded >30 μm) (Fig 2D and S2G, S2H and S2I Fig)
**III**	Fully hatched blastocysts in which all TE and ICM cells were hatched out of zone (Fig 2E and S3 Fig)

### Freezing and thawing

The biopsied blastocysts were vitrified using a Kitazato vitrification kit (Kitazato Biopharma) in combination with high-security vitrification straws (Cryo Bio System). The vitrification and thawing procedure was performed according to the manufacturer's instructions. Each blastocyst was stored in an individual straw.

### Frozen embryo transfer (FET) and luteal support

Blastocysts with the best morphological grading before biopsy were prioritized for thawing if their genetic testing results were found to be normal. A blastocyst was considered to be of high quality when the ICM and TE grades were both grade B or higher. Blastocysts were thawed using a commercially available warming solution (Kitazato Biopharma), according to the manufacturer's instructions. After thawing, the blastocysts were transferred to G2.5 medium and cultured for 1–2 h. Only blastocysts that survived the thawing and re-expansion process were considered suitable for transfer.

Each biopsied blastocyst was imaged by focusing on an equatorial plane of trophoblasts, the ICM, or ZP holes, before biopsy and embryo transfer. Digital images were saved to a compact disc and subsequently analyzed by the same senior embryologist to decide their morphological type. If a biopsied blastocyst changed its hatching type during 1–2 hours culture after thawing, the transferred patient was not included in this study.

Blastocysts were transferred either 5 days after ovulation in a natural cycle or 5 days after the initiation of progesterone (P) therapy with an endometrial preparation containing estradiol valerate and P. Briefly, 6 mg of estradiol valerate was administered from day 3 for 10–15 days, and luteal support was applied when satisfactory endometrial development (thickness ≥8mm) was confirmed by ultrasound examination.

### Pregnancy confirmation and follow-up

Clinical pregnancy was confirmed by the presence of a gestational sac and a fetal heartbeat at the 6-week ultrasound. Pregnant women with embryonic growth arrest or multifetal reduction after ultrasound confirmation of multiple pregnancies were defined as having a vanishing twin. Gestational age was calculated as the date of birth minus the date of embryo transfer plus 19. Preterm and post-term births were defined as deliveries before 37 or after 42 completed weeks of gestation. Neonates were categorized by birth weight: normal (2,500–4,000 g), very low birth weight (<2,500 g), and macrosomia (>4,000 g). As a part of the routine follow-up, couples were contacted by phone to get neonatal information, including their date of birth, birth weight, gender, birth defects, and neonatal diseases.

### Statistical analysis

Statistical analysis was carried out using Statistical Package for Social Sciences software version 19.0 (SPSS). Before analyses, a normality test was conducted for continuous variables. Normally distributed data were expressed as means±SD, and independent Student's *t* test (two groups) or analysis of variance (three groups) was used to compare the difference between the groups. Categorical variables were presented as percentages, the χ^2^ test was used for categorical variables, and Fisher's exact test was used if necessary. A P value of <0.05 was considered statistically significant.

## Results

### Relationship between blastocyst ICM/TE grade and hatching types

To analyze the incidence and morphological features of 8-shaped blastocysts with ICM incarceration, 10,730 biopsied blastocysts obtained from 2059 PGD/PGS patients were divided into three types (Table 2): 590 (5.5%) were type I (Fig 2A and S1 Fig), 4507 (42.0%) partially were type II (Fig 2B, 2C and 2D and S2 Fig), and 5633 (52.5%) were type III (Fig 2E and S3 Fig). After comparing ICM and trophectoderm (TE) grading of these blastocysts between groups, the percentage of grade A ICMs in type I blastocysts (22.2%) was comparable to that in type III blastocysts (20.1%) but higher than that in type II blastocysts (4.5%). Furthermore, the percentage of grade A TEs in type I blastocysts (4.2%) was comparable to that in type II (3.6%) but lower than that in type III blastocysts (13.5%).

**Table 2:** Relationship between ICM/TE grade and hatching types in PGD/PGS blastocysts.

Type	I	II	III	P value		
**No. of blastocysts**	590	4507	5633	Type I vs. II	Type I vs. III	Type II vs. III
**ICM grading**				< 0.001	0.226	< 0.001
**A**	131(22.2)	203(4.5)	1132(20.1)			
**B**	459(77.8)	4304(95.5)	4501(79.9)			
**TE grading**				0.538	< 0.001	< 0.001
**A**	25(4.2)	162(3.6)	761(13.5)			
**B**	362(61.4)	2709(60.1)	3729(66.2)			
**C**	203(34.4)	1636(36.3)	1143(20.3)			

Note: Values in parentheses are percentages.

type I: 8-shape blastocysts with ICM incarceration; type II: partially hatched blastocysts without ICM incarceration; type III: fully hatched blastocysts

### Patient characteristics

Table 3 presents the clinical and demographic characteristics of the patients in these groups. There were no significant differences between the groups in terms of maternal age, BMI, infertility period, infertility types, treated cycles, patient types, endometrial preparation protocols, and median endometrial (EM) thickness.

**Table 3:** Characteristics of PGD/PGS patients transferred a single blastocyst with different hatching types.

Group	I	II	III
**No. of cycles**	70	353	569
**Female age (yr)**	31.5±4.4	31.1±4.6	30.6±4.4
**<35**	57(81.4)	276(78.2)	459(80.4)
**35–38**	8(11.4)	50(14.2)	79(13.9)
**>38**	5(7.1)	27(7.6)	31(5.4)
**Body mass index (kg/m**^**2**^**)**	22.1±2.5	21.7±2.9	21.5±2.7
**<18.5**	6(8.6)	28(7.9)	62(10.9)
**18.5–24.9**	55(78.6)	287(81.3)	448(78.7)
**≥25**	9(12.9)	38(10.8)	59(10.4)
**Infertility period (yr)**	3.8±2.8	3.9±3.2	3.5±2.7
**Infertility types**			
**Primary**	22(31.4)	108(30.6)	171(29.9)
**Secondary**	48(68.6)	245(69.4)	400(70.1)
**Treated cycles**			
**≥2**^**nd**^ **cycle**	20(28.6)	91(25.8)	122(21.4)
**Patients type**			
**PGD**	58(82.9)	305(86.4)	487(85.6)
**PGS**	12(17.1)	48(13.6)	82(14.4)
**Endometrium (EM) preparation**			
**NC**	26(37.1)	117(33.1)	207(36.4)
**HRT**	44(62.9)	229(64.9)	362(63.6)
**EM thickness (mm)**	11.8±2.0	11.5±1.9	11.6±1.8

Note: Values in parentheses are percentages.

Group I: patients transferred an 8-shape blastocyst with ICM incarceration; Group II: patients transferred a partially hatched blastocyst without ICM incarceration; Group III: patients transferred a fully hatched blastocyst

### Clinical outcomes

There were no significant differences between patients in groups I, II, and III in terms of percentage of high-quality blastocysts (64.3%, 58.9%, and 65.9%, respectively), clinical pregnancy rate (61.4%, 55.8%, and 58.7%, respectively), percentage of fetal hearts (62.9%, 58.1%, and59.9%, respectively), MZT pregnancy rate (2.3%, 4.1%, and 2.3%, respectively), miscarriage (20.9%, 19.3%, and 16.5%, respectively), ectopic pregnancy rate (0%, 1.5%, and 1.2%, respectively), percentage of vanishing twins (0%, 1%, and 0.6%, respectively), percentage of cesarean section (79.4%, 74.4%, and 78.5%, respectively), live birth rate (48.6%, 44.2%, and 48.0%, respectively), and MZT birth rate (0%, 1.3%, and 0.7%, respectively; Table 4).

**Table 4:** Clinical outcomes of PGD/PGS patients transferred a single blastocyst with different hatching types.

Group	I	II	III
**Transferred blastocysts**	70	353	569
**High-quality blastocysts**	45(64.3)	208(58.9^)^	375(65.9)
**Clinical pregnancy**	43(61.4)	197(55.8)	334(58.7)
**Fetal hearts**	44(62.9)	205(58.1)	341(59.9)
**MZT pregnancy**	1(2.3)	8(4.1)	7(2.1)
**Miscarriage**	9(20.9)	38(19.3)	55(16.5)
**≤12 weeks**	8(18.6)	34(17.3)	47(14.1)
**>12 weeks**	1(2.3)	4(2.0)	8(2.3)
**Ectopic pregnancy**	0	3(1.5)	4(1.2)
**Vanishing twin**	0	2(1.0)	2(0.6)
**Cesarean section**	27(79.4)	116(74.4)	215(78.5)
**Live births**	34(48.6)	156(44.2)	274(48.2)
**MZT birth**	0	2(1.3)	2(0.7)

Note: Values in parentheses are percentages.

Group I: patients transferred an 8-shape blastocyst with ICM incarceration; Group II: patients transferred a partially hatched blastocyst without ICM incarceration; Group III: patients transferred a fully hatched blastocyst

In artificial insemination patients, clinical pregnancy, MZT pregnancy and MZT birth rate were 24.0% (3829/15939), 0.34% (13/3829) and 0.34% (13/3829), respectively. The MZT pregnancy rate in AI patients was significantly lower than that in PGD/PGS patients (2.8%, 16/574).

### Neonatal outcomes

A total of 34, 158, and 276 infants were born in groups I, II, and III, respectively. One perinatal death occurred in Group II. There were no significant differences in gender ratio (percentage of boys, 52.9% vs. 58.9% vs. 62.3%), median gestational age (38.8 ± 1.7 vs. 38.8 ± 1.7 vs. 38.9 ± 1.7 weeks), frequency of preterm birth (8.8% vs. 7.1% vs. 7.7%), median birth weight (3.38 ± 0.50 vs. 3.42 ± 0.55 vs. 3.42 ± 0.53 kg), percentage of low birth weight infant (5.9% vs. 4.4% vs. 2.9%), incidence of macrosomia (8.8% vs. 9.5% vs. 10.9%), and percentage of normal births (94.1% vs. 95.6% vs. 98.6%) between the three groups.

One newborn from Group I had patent oval foramen and one suffered polydactyly; In Group II, there was one case of patent oval foramen, one case of patent oval foramen combined with atrial septal defect, one case of patent ductus arteriosus, one case of hypodactyly, one case of congenital hip dysplasia, and one case of hearing impairment. In Group III, there were two cases of cheilopalatognathus, one case of patent oval foramen, and one case of congenital heart disease, but the rate of birth defects did not significantly differ between the groups (5.9% vs. 3.8% vs. 1.4%; Table 5). None of the MZT newborns had birth defects.

**Table 5:** Neonatal outcomes of PGD/PGS patients transferred a single blastocyst with different hatching types.

Group	I	II	III
**No. of newborns**	34	158	276
**singleton**	34(100)	154(97.5)	272(98.6)
**twin**	0(0)	4(2.5)	4(1.4)
**Sexual ratio** **(Male/female)**	1.13	1.43	1.65
**Male**	18(52.9)	93(58.9)	172(62.3)
**female**	16(47.1)	65(42.2)	104(37.7)
**Gestational age (wk)**	38.8±1.7	38.8±1.7	38.9±1.7
**37~42wk**	31(91.2)	145(92.9)	252(92.0)
**<37wk**	3(8.8)	11(7.1)	21(7.6)
**>42wk**	0	0	1(0.4)
**Birth weight (kg)**	3.38±0.50	3.42±0.55	3.43±0.53
**2.5~4.0 kg**	29(85.3)	136(86.1)	238(86.2)
**<2.5 kg**	2(5.9)	7(4.4)	8(2.9)
**>4.0 kg**	3(8.8)	15(9.5)	30(10.9)
**Normal birth**	32(94.1)	151(95.6)	272(98.6)
**Neonatal mortality**	0	1(0.6)	0
**Birth defect**	2(5.9)	6(3.8)	4(1.4)

Note: Values in parentheses are percentages.

Group I: patients transferred an 8-shape blastocyst with ICM incarceration; Group II: patients transferred a partially hatched blastocyst without ICM incarceration; Group III: patients transferred a fully hatched blastocyst

## Discussion

In this study, we compared the clinical and neonatal outcomes in PGD/PGS patients who received a single blastocyst transfer with different hatching types. This study does not support the previous hypothesis that ICM splitting by a small ZP opening can potentially cause MZT in humans. The results of the present study suggested that ICM incarceration in 8-shaped blastocysts does not increase the incidence of MZT; furthermore, it does not have any negative effects on newborns.

The mechanism of MZT in humans is unknown: the timing of MZT is probably not fixed and the mechanism varies from one to another [3]. A widely accepted model of MZT is based on the hitherto unproven hypothesis of postzygotic division of the conceptus [18]. In this model, the number of fetuses, chorions, and amnions are determined by the time at which the embryo splits [18].

Several theories have been proposed to explain the increased rate of MZT observed after ART [19]. These include ovarian stimulation [20], manipulation of the ZP [21], hardening of the ZP due to extended in vitro culture [22], changes in the culture medium [23, 24] and multiple pregnancies [25]. Some authors hypothesize that a breach or hardening in the ZP may induce ICM splitting by a small zone hole, resulting in MZT. However, this concern has been refuted by several recent studies [10, 19, 26, 27].

In this study, we investigated the relationship between blastocyst grading and their hatching types. The results revealed that the ICM/TE grades contributes to ICM incarceration. Compared to partially and fully hatched blastocysts, 8-shaped blastocysts with ICM incarceration showed relatively higher ICM and lower TE grades. Conventionally, a ZP hole is made in day 3 embryos before blastocyst biopsy, which is similar to the procedure of AH. This manipulation changes the hatching procedure: after AH, ZP thinning does not occur, and TE cells herniate out of the ZP opening when the blastocoels begin to expand. If the size of the ICM exceeds the inner diameter of the ZP opening, it may become trapped when passing through the ZP hole. Furthermore, limited TE cells may not expand the ZP hole further, allowing the blastocyst to fully hatch out. In our study, we found that 20.1% of all blastocysts grade A ICM could fully hatch out. There may be two reasons for this: first, the ICMs of these blastocysts may pass through ZP holes at an early stage when they are not too large in size, and the ICM cells continue to proliferate to reach grade A after the blastocyst fully hatches out. Second, these blastocysts showed a higher TE grade, and these TE cells may expand the ZP hole further, enabling the blastocyst to fully hatch out.

Previous studies used OCT4 staining to confirm ICM splitting in 8-shaped blastocysts in mice [14] and humans [15]. Unfortunately, we did not identify whether ICM incarceration in a small zona hole resulted in its splitting. The similar MZT pregnancy rates observed in the 8-shaped, partially hatched, and fully hatched blastocysts suggested that ICM incarceration does not induce permanent ICM splitting. In this study, 25- to 30-μm zona holes could not produce a cellular bridge narrow enough to split the ICM or cause the blastocyst to break. In mammals, blastocyst hatching *in vivo* differs from that *in vitro* [28] [29]. *In vitro*, blastocyst hatching occurs as a result of the tension of the periodic contraction and expansion and enzymatic digestion of trophoblasts [30, 31]; while *in vivo* hatching occurs following interaction between blastocyst and uterus [32]. Intrauterine ZP lytic activity during the preimplantation period may help blastocysts escape the ZP [32]. It has been demonstrated in many species that the blastocyst does not normally hatch but the zona dissolves due to proteinase activity [33]. Thus, ICMs trapped in the zona hole may be released and recovered after the ZP lysis.

In this study, the incidence of birth defects did not differ between the groups, and no birth defect occurred in MZT newborns. The results suggested that ICM incarceration by a small zona hole does not induce damage to ICM cells. Furthermore, we should remember that PGD/PGS may help in selection of a healthy embryo. However, we found a high miscarriage rate associated with MZT pregnancy in this study. A total of 16 MZT pregnancies in three groups only resulted in 4 MZT live births and 4 singleton live births (vanishing twin). The remaining 20 of 32 embryos (62.5%) from MZT pregnancies did not develop to term and had to be aborted in the first trimester. This is consistent with previous reports that MZT results in a very high rate of spontaneous abortions and fetal abnormalities [34, 35].

Verpoest et al. [36] reported that the incidence of MZT was not increased in PGD (1.5%) compared with regular ICSI with blastocyst transfer (2.1%), and the authors suggested that ZP breaching and biopsy may not contribute to ICM splitting and MZT. Scott Sills et al. [37] suggested that the causal relationship between AH and MZ twinning is highly speculative: among IVF study patients, they found that the frequency of MZT did not significantly differ between manipulated and intact ZP subgroups. Other studies have not been able to establish a relationship between ZP breaching techniques and MZT [23, 38, 39]. In this study, zona-free blastocysts resulted in an MZT rate of 2.1%, which is similar to that observed with 8-shaped blastocysts (2.3%). Consistent with this result, complete removal of the ZP by pronase digestion prior to blastocyst embryo transfer did not eliminate monozygotic pregnancies following IVF [40]. Thus, even if the zona may be involved in some cases of MZT, it is unlikely to be an exclusive mechanism.

In this study, the overall MZT pregnancy rate per established clinical pregnancy was 2.8% (16/574), which appeared higher than the rate natural conception. A meta-analysis and a recent large study did not show any associations between ICSI or AH and MZT[10, 19]. Blastocyst culture appears to be one of the most important factors contributing to MZT pregnancies [7, 10, 41], but not all publications support this association [27, 42]. Thus, the mechanism of MZT in ART remains to be elucidated.

One of the strengths of this study was that only cycles with single blastocyst transfer were included. Although DNA fingerprinting is the gold standard to determine MZT, the incidence of MZT is also reliable in this study. However, there are four limitations in this preliminary study that may have affected the results. First, the blastocysts were classified according to their hatching status, identified on the basis of their morphological features. The ICM features were not confirmed by immunofluorescence staining. Second, this is a retrospective study. The factors between the study and control groups are confounding. For example, the morphology of blastocysts transferred is mixed in Group II, and included blastocysts with ICM inside and outside the ZP holes or U-shape hatched blastocysts; however, we did not subgroup these cases as that would have reduced the sample size, and a tentative analysis revealed no differences. Third, the sample size of this study is limited. The transferred blastocysts only represent a small number of samples in the cohort, which may introduce bias due to sampling error. A larger volume of data may be obtained in a multicenter study. Fourth, the follow-up period was limited. Further follow-up should be conducted to collect data from these children in childhood, puberty, and adulthood. In the future, we expect a multicenter randomized controlled trial with larger sample size to verify the findings of this study.

Because of the increased risk and healthcare costs associated with MZT following ART, it is important to decipher the reasons underlying the higher incidence of MZT in the setting of ART and to identify approaches to reduce this incidence[43]. Although we still cannot explain the cause of MZT after ART, this study validates existing data regarding the possible causes of MZT following ART and provides useful information for counseling patients undergoing PGD/PGS about the potential risks of ART techniques.

## Conclusions

The results revealed that the ICM/TE grades contributes to ICM incarceration. Compared to partially or fully hatched blastocysts, 8-shaped blastocysts with ICM incarceration showed relatively higher ICM or lower TE grades, respectively. The results do not support the hypothesis that splitting of the ICM through a small zona hole may result in MZT. Inner cell mass incarceration in figure 8-shaped blastocysts does not increase monozygotic twinning and has no negative effects on newborns in preimplantation genetic diagnosis and screening patients.

## Supporting information

S1 Fig Type I blastocysts. Figure 8-shaped blastocysts with ICM incarceration in which a part of TE cells were hatched out, but ICMs were trapped in the ZP opening (zona hole ≤ 30 μm); Bar = 30 μm.
(TIFF)

S2 Fig Type II blastocysts. Partially hatched blastocysts without ICM incarceration in which ICMs were inside (A, B and C) or outside (D, E and F) of the ZP opening, or the blastocysts hatched with a U-shape (zona hole expanded >30 μm) (G, H and I); Bar = 30 μm.
(TIFF)

S3 Fig Type III blastocysts. Fully hatched blastocysts in which all TE and ICM cells were hatched out of zone; Bar = 30 μm.
(TIFF)
