# Supplementary figures and images for: Inner cell mass incarceration in 8-shaped blastocysts does not increase monozygotic twinning in preimplantation genetic diagnosis and screening patients
Source: PLoS One. 2018 Jan 9;13(1):e0190776. doi: 10.1371/journal.pone.0190776 (PMC5760060; doi:10.1371/journal.pone.0190776)

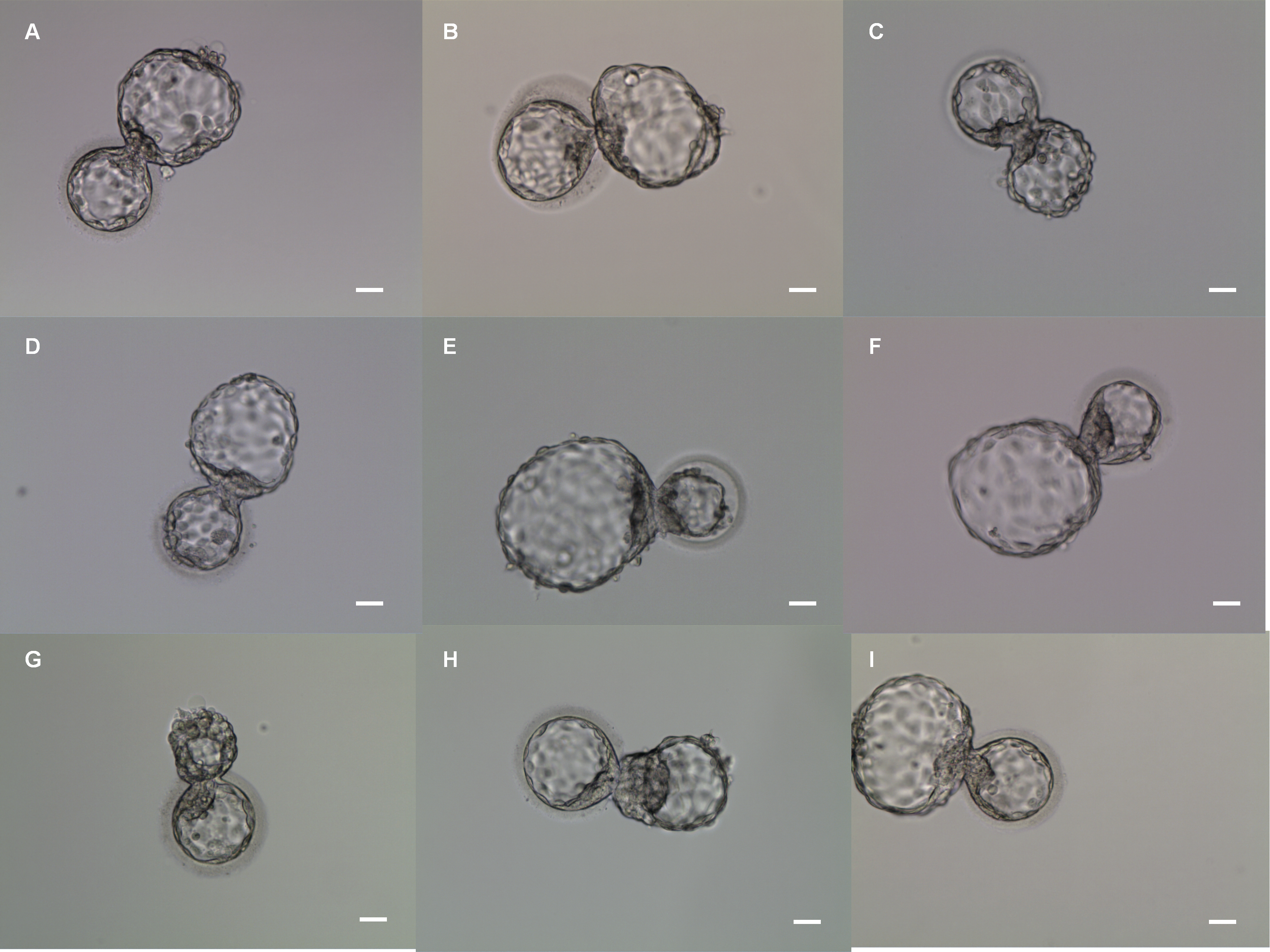

Supplement: S1 Fig — Figure 8-shaped blastocysts with ICM incarceration in which a part of TE cells were hatched out, but ICMs were trapped in the ZP opening (zona hole ≤ 30 μm); Bar = 30 μm. (TIFF) [file pone.0190776.s001.tiff]

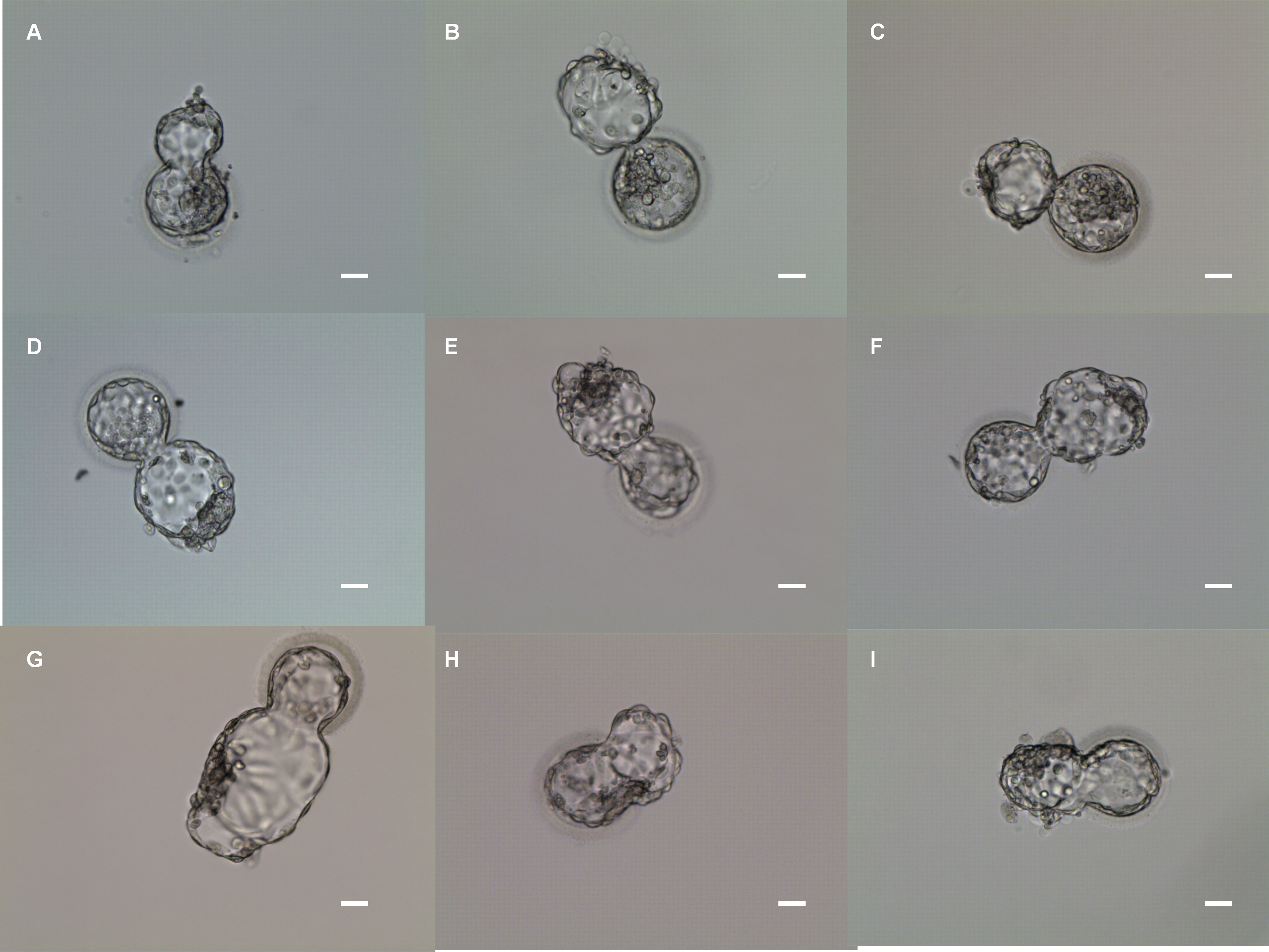

Supplement: S2 Fig — Partially hatched blastocysts without ICM incarceration in which ICMs were inside (A, B and C) or outside (D, E and F) of the ZP opening, or the blastocysts hatched with a U-shape (zona hole expanded >30 μm) (G, H and I); Bar = 30 μm. (TIFF) [file pone.0190776.s002.tiff]

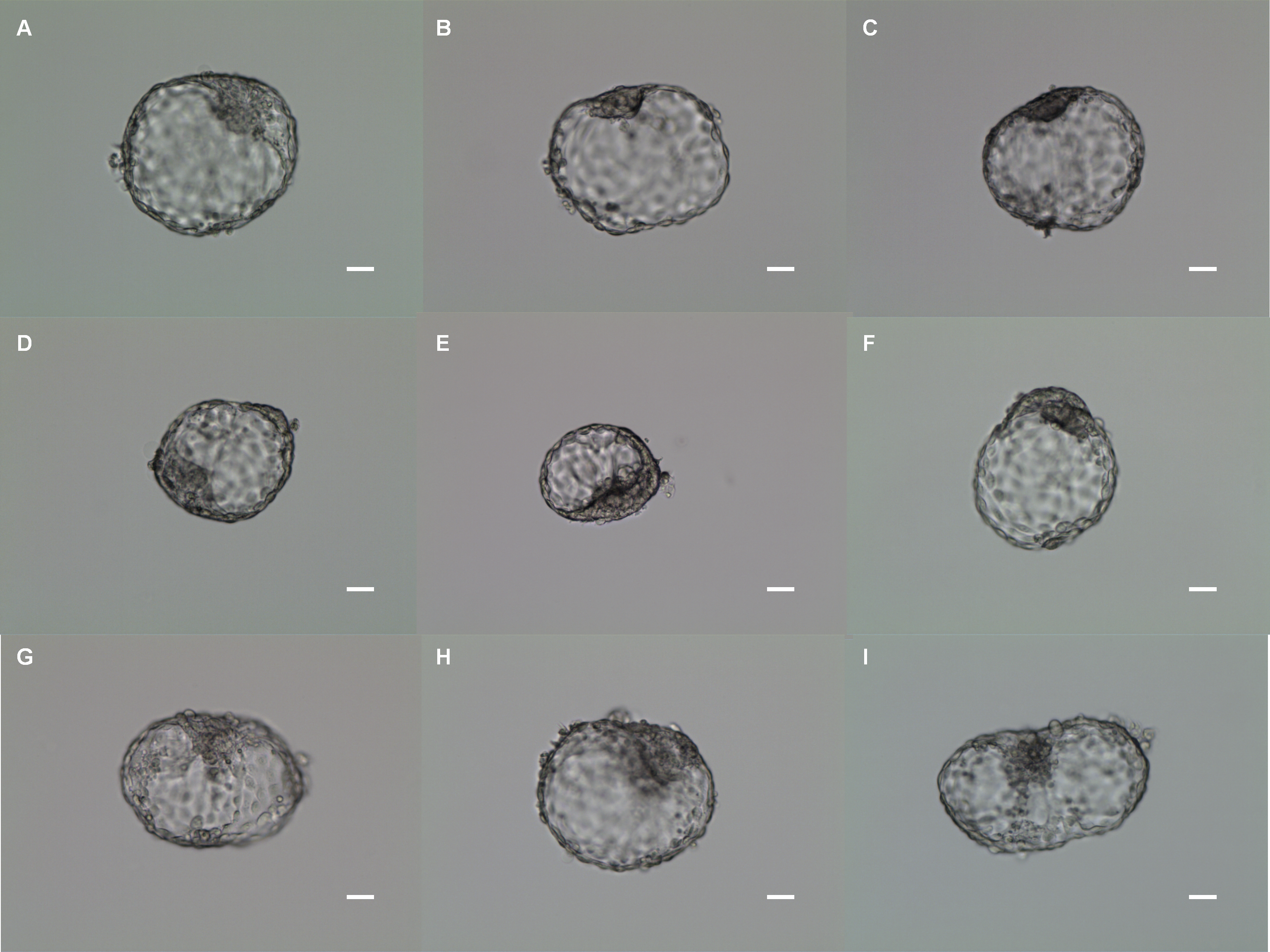

Supplement: S3 Fig — Fully hatched blastocysts in which all TE and ICM cells were hatched out of zone; Bar = 30 μm. (TIFF) [file pone.0190776.s003.tiff]
